# Clinical Use of Paraprobiotics for Pregnant Women with Periodontitis: Randomized Clinical Trial

**DOI:** 10.3390/dj12040116

**Published:** 2024-04-19

**Authors:** Andrea Butera, Maurizio Pascadopoli, Maria Gloria Nardi, Chiara Ogliari, Alessandro Chiesa, Camilla Preda, Giulia Perego, Andrea Scribante

**Affiliations:** 1 Unit of Dental Hygiene, Section of Dentistry, Department of Clinical, Surgical, Diagnostic and Pediatric Sciences, University of Pavia, 27100 Pavia, Italy; 2 Unit of Orthodontics and Pediatric Dentistry, Section of Dentistry, Department of Clinical, Surgical, Diagnostic and Pediatric Sciences, University of Pavia, 27100 Pavia, Italy; 3 Policlinico San Matteo, 27100 Pavia, Italy

**Keywords:** periodontal disease, gingivitis, pregnant women, pregnancy, paraprobiotics

## Abstract

Periodontal disease is very common in pregnant women. Paraprobiotics are a subset of probiotics. They can be defined as inactivated microbial cells providing health benefits to the host and are considered particularly safe. The aim of this study was to compare the periodontal health of pregnant women and puerperae after 6 months of home use of paraprobiotics. A total of 30 pregnant women were enrolled and divided into two groups: the test group, who had to use a paraprobiotic-based toothpaste (Biorepair Peribioma Pro, Coswell S.p.A., Funo di Argelato, BO, Italy) and mousse (Mousse Mouthwash Biorepair Peribioma, Coswell S.p.A.) twice a day, and the control group, who had to use only the paraprobiotic-based toothpaste. The time frames of the study were: 1 month (T1), 3 months (T2) and 6 months (T3), and data were collected during pregnancy and in the period immediately following delivery. The following indices were evaluated at T0, T1, T2 and T3: clinical attachment loss (CAL), probing pocket depth (PPD), bleeding on probing (BOP), plaque control record (PCR), modified marginal gingival index (mMGI), papillary marginal gingival index (PMGI) and recessions (R). All data were subjected to statistical analysis. PCR decreased significantly from T0 to T1 in the control group and from T0 to T2 and from T0 to T3 in the test group. BOP tended to decrease in both groups, but a significant reduction was observed only in the test group. CAL, PPD, PMGI and mMGI tended to decrease gradually in both groups without significant differences between or within groups. The combination of the paraprobiotic-based toothpaste and the paraprobiotic-based mousse significantly reduced BoP and plaque control over time, although there were no significant differences with the use of the paraprobiotic-based toothpaste alone. In addition, the combination of the two products promoted a trend towards the better stabilization of recessions.

## 1. Introduction

Periodontal disease is one of the most common chronic multifactorial diseases worldwide [1,2], affecting the alveolar bone and connective tissues that surround and support the teeth [3]. The disease initially presents as gingivitis, which is associated with bleeding, swelling and pain in the gums. If left untreated, it can progress to periodontitis, which is characterized by loss of periodontal attachment and bone resorption [4], while in the most severe cases it can lead to tooth loss [5]. Bacteria have been observed to colonize tooth surfaces and form dental plaque, which can induce tissue inflammation through cellular activation and the production of pro-inflammatory cytokines [6,7]. Smoking is one of the major risk factors: a higher presence of periodontal pathogens can be found in smokers. However, smoking negatively affects host immune cells and reduces the immune response [8]. Another important risk factor is diabetes: it has been scientifically proven that uncontrolled diabetes increases the risk of developing periodontal disease by altering connective tissue metabolism and the immune and inflammatory response [9]. According to a recent systematic review and meta-analysis, periodontal disease is highly prevalent in pregnant women and may be associated with poorer quality of life, systemic disease and adverse pregnancy outcomes [10]. Although there is no consensus in the literature, periodontal disease has been reported to be associated with adverse pregnancy outcomes such as preterm birth, low birth weight and pre-eclampsia [11]. It is estimated that one in five pregnant women has periodontal disease [12]. Pregnancy is associated with an increased risk of gingivitis due to higher levels of estrogen and progesterone [13]. Basavaraju et al. observed a selective growth of *Prevotella intermedia*, *Porphyromonas gingivalis* and *Tannerella* species in pregnant women, probably because progesterone is a nutrient source for these bacteria [14]. Gingivitis due to plaque accumulation may indeed be aggravated by these endogenous steroid hormones [15]. Sexual hormone alterations in pregnant women are responsible for reduced immune response to bacteria and gingival vascular changes that promote gingivitis [16]. Plaque index and education level have been shown to be inversely related in pregnant women, suggesting the need for educational programs for women to better manage periodontal health during pregnancy [17]. Geurs et al. showed that oral health education can be effective in reducing gingival inflammation in pregnant women [18]. In addition, it is important to educate pregnant women about good oral health [19] and the oral health of their children [20]. Some studies in the literature have also demonstrated the effectiveness of therapeutic measures to improve oral hygiene and periodontal health in pregnant women [21,22]. Probiotics are live microorganisms conferring healthy benefits on the host, whether administered appropriately [23]. Several studies in the literature demonstrate the benefits of a probiotic-supported therapy on periodontal disease [24]. It has been observed that probiotics play a role in the prevention of many diseases in pregnant women, such as gestational diabetes [25,26], mastitis [27], constipation [28], post-partum depression [29] and Group B Streptococcus bacteria proliferation [30]. Some studies also suggest a protective action towards preeclampsia [31], vaginal infections [32], maternal and infant weight gain [33] and allergic diseases [34]. According to a systematic review and meta-analysis, probiotics do not endanger the mother and infant’s health during or after pregnancy or during lactation [35]. Paraprobiotics are a subset of probiotics, they can be defined as inactivated microbial cells (non-viable) providing a health benefit to the host. Because of their ability to regulate the immune system and their anti-inflammatory, antiproliferative and antioxidant properties, they could be considered safer in at-risk patients, such as elderly and immunocompromised patients [36]. It has been shown that paraprobiotics combined to the non-surgical scaling and root-planing (SRP) promote a major reduction in bacteria involved in periodontal disease and result in a significant reduction in many periodontal indexes [37]. There is little evidence about the effects of probiotics and, particularly, paraprobiotics on pregnant women’ periodontal health. Therefore, the aim of the present study is to compare the periodontal health status of pregnant women and puerperae treated with a paraprobiotic-based toothpaste and mousse twice daily, with the use of the paraprobiotic-based toothpaste alone after 1 month (T1), after 3 months (T2) and after 6 months (T3). The null hypothesis is that there are no significant differences between the two treatments with respect to variables related to periodontal health.

## 2. Materials and Methods

### 2.1. Study Design

This was a randomized clinical trial (RCT) with two parallel arms, each with an equal number of participants. The Declaration of Helsinki was followed for ethical concerns and approval was granted by the Unit Internal Review Board (n°: 2022-0316). The CONSORT statement was followed for the conduct and writing of the study, and the protocol was registered on clinicaltrials.gov (NCT: NCT05400538). Patients signed the informed consent prior to the start of the trial. Enrolment began in June 2022, and the entire study ended in October 2023.

### 2.2. Participants

In total, 30 pregnant patients attending the Department of Obstetrics and Gynaecology, Fondazione IRCCS Policlinico San Matteo Pavia, and scheduled for routine periodontal care at the Department of Dental Hygiene, Section of Dentistry, Department of Clinical, Surgical, Diagnostic and Paediatric Sciences, University of Pavia, 27100 Pavia, Italy, were recruited for the study. The inclusion criteria were as follows: age 18–35 years, women at least at the fourth month of pregnancy, periodontitis (stage I, grade B). The exclusion criteria were as follows: the presence of a cardiac pacemaker, neurological and psychiatric diseases, patients taking bisphosphonates in the last 12 months before the start of the study, patients undergoing anticancer therapy, poor compliance. The main characteristics of the participants (pregnancy period at the enrollment, previous pregnancies and caesarean delivery) are summarized in Table 1 and Table 2.

### 2.3. Interventions and Outcomes

At the first visit (T0), patients were examined with a periodontal probe (UNC Probe 15; Hu-Friedy, Chicago, IL, USA) and the following indices were assessed on each tooth: clinical attachment loss (CAL) [38]; probing pocket depth (PPD) [38]; bleeding on probing (BOP) [39], plaque control record (PCR) [40]; modified marginal gingival index (mMGI) [41]; papillary marginal gingival index (PMGI) [42]; and recession (R) [38]. Clinical examinations were performed by a calibrated operator. Calibration upon intra-examiner repeatability for CAL was considered valid with a percentage of agreement within ±2 mm between repeated measurements of at least 98% [43].

An operator not involved in the clinical procedures randomized the patients into two groups:

The test group, in which patients had to use a paraprobiotic-based toothpaste (Biorepair Peribioma Pro, Coswell S.p.A.) (Table 2) and mousse (Mousse Mouthwash Biorepair Peribioma, Coswell S.p.A.) twice a day for routine oral care at home;

A control group in which patients were required to use only the paraprobiotic-based toothpaste (Biorepair Peribioma Pro, Coswell S.p.A.) (Table 3) twice daily for routine home oral care. Patients were given oral instructions on how to correctly use the products provided. They also received an informative booklet together with informed consent.

Patients were assessed at 1 month (T1), 3 months (T2) and 6 months (T3), and data were collected during pregnancy and in the period immediately following delivery. At each time frame, patients were asked and they confirmed that they ha used the products according to the instructions provided. The present study was designed as a superiority RCT, as it aims to evaluate the effectiveness of the combined treatment (toothpaste + mouthwash) with that of the single treatment (toothpaste).

### 2.4. Sample Size

With a type I error alpha = 0.05 and type II error power = 80%, considering two independent study groups, the sample size calculation was performed using “clinical attachment level (CAL)” as the primary outcome. The following formula was used:

$$Sample\;size=\frac{Z_{\left(1-\frac{\alpha }{2}\right)}^{2}p\left(1-p\right)}{d^{2}}$$

where p is the expected proportion in population, $z_{\left(1-\frac{\alpha }{2}\right)}$ is the standard normal variate corresponding to 1.96 with a type I error of 5% and d is the confidence level chosen. The study of Yarkac et al. [44] was used to calculate the sample size of the present study: an expected value of 1.93 was hypothesized, with an expected difference of 0.3 and a standard deviation of 0.29. Therefore, 15 patients per group (30 patients in total) were required to conduct the study. Considering the possibility of losing patients at follow-up, sample size can be calculated as follows: if n is the sample size required as per formula and if d is the drop-out rate, then adjusted sample size N1 is obtained as: N1 = n/(1 − d). Considering a drop-out rate of 10%, N1 is equivalent to 17 patients per group (34 patients in total).

### 2.5. Randomization and Blinding

A block randomization table was used for patient allocation, with a random sequence provided by the data analyst, taking into account a permuted block of 30 total participants. One staff member, who had not been involved in the previous procedures, enrolled and administered the professional verbal procedures and recorded the results. Using pre-prepared, sequentially numbered, opaque, sealed envelopes (SNOSE), an assistant assigned participants to their respective groups, concealing the products intended for home use. The data analyst was unaware of the allocation and results. The home oral hygiene products were hidden. Both the dentist and the patients were unaware of the treatment administered.

### 2.6. Statistical Analysis

Data were analyzed using R software (R version 3.1.3, R Development Core Team, R Foundation for Statistical Computing, Vienna, Austria). Descriptive statistics were calculated as mean, standard deviation, median, minimum, maximum. Inferential statistics were performed. The normality of the data was assessed using the Kolmogorov–Smirnov test. Friedman’s test for repeated measures was used, followed by Dunn’s post hoc test. For all statistical tests, significance was set at *p* < 0.05.

## 3. Results

### 3.1. Participants Flow and Baseline Data

**Figure 1:**
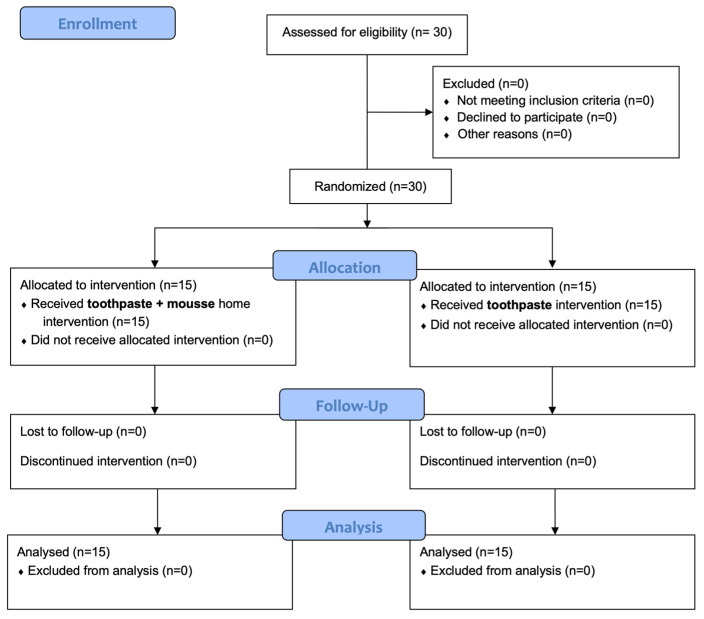
CONSORT flow chart.

Figure 1 shows the flow chart of the study. In total, 30 patients were enrolled and all completed the study.

At baseline, the demographics of the study sample showed a mean age of 30.17 ± 30.03 years, with a mean age of 28.33 ± 3.02 for the control group and 31.4 ± 2.36 for the test group.

The descriptive and inferential statistics for the study variables are presented using a letter-based presentation [45].

### 3.2. Clinical Attachment Loss (CAL)

The CAL scores are shown in Table 4. A decrease can be seen from T0 to T3, but with no significant differences between or within groups (*p* > 0.05).

### 3.3. Probing Pocket Depth (PPD)

The PPD scores are shown in Table 5. The results of the study show that there were no significant comparisons between or within groups at any time point (*p* > 0.05).

### 3.4. Plaque Control Record (PCR)

The PCR results are shown in Table 6. In the control group, a significant difference was found for T0-T1 (*p* < 0.05), with no significant differences for the other time frames (*p* > 0.05). In the test group, significant differences were found at T0-T2 and T0-T3 (*p* < 0.05). There were no significant differences between the two groups (*p* > 0.05).

### 3.5. Bleeding on Probing (BOP)

BOP scores are shown in Table 7 and show a decrease from T0 to T3 in both groups. In terms of between-group differences, no significant differences were found for the control group (*p* > 0.05), while significant differences were found for T0-T2 and T0-T3 in the test group (*p* < 0.05). No significant differences were found between the two groups (*p* > 0.05).

### 3.6. Modified Marginal Gingival Index (mMGI)

The mMGI scores are shown in Table 8. No significant between- or within-group differences were found between the two study groups (*p* > 0.05), but a gradual decrease can be seen in both groups.

### 3.7. Papillary Marginal Gingival Index (PMGI)

The PMGI scores are shown in Table 9. A gradual decrease can be seen in the control group, whereas a milder decrease can be seen in the test group. No significant between- or within-group differences were found between the two study groups (*p* > 0.05).

### 3.8. Recession (R)

The R values are shown in Table 10. In the control group, increasing values are found, whereas stable values are found in the test group. No significant inter- or intragroup differences were found between the two study groups (*p* > 0.05).

### 3.9. Harms and Adverse Effects

No harm or adverse effects occurred during the trial.

## 4. Discussion

During pregnancy, women undergo physical, hormonal and emotional changes that can significantly affect their quality of life [46]. Periodontal disease has been shown to have a negative impact on patients’ quality of life [47]. In fact, effective periodontal treatment during pregnancy has been shown to result in a statistically significant improvement in oral-health related quality of life (OHRQoL) [48]. Unfortunately, there is a lack of knowledge in this area: Togoo et al., in their cross-sectional questionnaire-based study, found that most pregnant women were unaware of the prevention, causes, consequences and treatment of gingivitis in pregnancy [49]. Educational programs for pregnant women provided by oral health professionals should be developed [50]. Liu et al. showed that oral health education booklets, when combined with oral hygiene instruction, individualized feedback and suggested solutions to overcome self-care barriers, were more effective in reducing visible plaque index (VPI) and BOP in pregnant women [51]. If not associated with periodontal attachment loss, gingival changes during pregnancy may be transient and often resolve after delivery [16]. Gingival inflammation usually increases from 16 to 40 weeks’ gestation and then decreases after delivery [52]. Women have been shown to undergo metabolic, hormonal and immunological changes during pregnancy, which may also affect their oral microbiome [53]. Increased proliferation of some bacterial taxa, such as *Lactobacillus* and *Streptococcus*, has been observed [54]. When combined with poor oral hygiene, this can lead to a significant risk of developing periodontal disease [55]. Oral microorganisms can reach the fetal placental unit via both the bloodstream and the genitourinary tract and periodontal inflammatory mediators can affect the fetal placental unit directly or indirectly by reaching the liver and promoting systemic inflammation [56]. Various probiotics have been developed and tested in pregnant women to improve their periodontal health. Gadzhula et al. analyzed the effects of a hyaluronate-based gel (Gengigel Gingival Gel, Ricerfarma s.r.l., Milan, Italy) and *L. reuteri* probiotic lozenges (Prodentis, BioGaia, Stockholm, Sweden) on pregnant women. Gengigel Gingival Gel should be applied twice a day for 2–3 min, and the *L. reuteri* probiotic Prodentis should be taken sublingually 20 times in each trimester. It has been shown that hyaluronic acid is effective in reducing inflammatory levels by improving the metabolism of non-sulphated glycosaminoglycans, while probiotic *L. reuteri* Prodentis prevents oral biofilm colonization and reduces the risk of disease [21]. A simpler protocol was proposed by Erchick et al. who compared the periodontal outcomes of three alcohol-free antiseptic oral rinses used twice daily for 12 weeks by pregnant Nepalese women. Unlike cetylpyridinium chloride-based or sodium chloride-based rinses, the chlorhexidine-based rinse significantly reduced gingivitis rates and BOP extension in the sample. However, antiseptic mouthrinses can play a complementary and not an exclusive role in oral hygiene procedures [57]. Kraivaphan et al. studied the effects of a triclosan/copolymer toothpaste on 180 women in their third trimester of pregnancy. The women were asked to brush twice a day for one minute with the toothpaste for five months. The results were compared with a placebo control group. The results showed that the triclosan/copolymer toothpaste reduced plaque by 40.5%, gingivitis by 22.5% and gingival bleeding by 35.3% [58]. According to Riley and Lamont, triclosane/copolymer toothpaste, which must be combined with fluoride, moderately reduces gingival inflammation and bleeding, but there is not enough evidence for its role in preventing periodontitis [59]. Tecco et al. analyzed the results of the daily use of ozonated water in conjunction with oral hygiene on pregnant women from the 14th to the 30th week of pregnancy affected by gingivitis. Plaque index (PI) and bleeding on probing (BOP) were assessed and the results were compared with a control group that only performed oral hygiene. It was shown that pregnant women using ozonated water experienced a more significant reduction in gingival bleeding, suggesting beneficial antimicrobial properties of ozonated water. However, ozonated water has to be combined with other oral hygiene products and the periodontal health status of the enrolled women was evaluated using only two parameters (PI and BOP) [60]. The present study aims to compare the periodontal health status of pregnant women using only a paraprobiotic-based toothpaste (Biorepair Peribioma Pro) and the paraprobiotic-based toothpaste (Biorepair Peribioma Pro) together with the mousse (Mousse Mouthwash Biorepair Peribioma). Unlike other products tested, these paraprobiotics are easy to use and play a fundamental role in daily oral hygiene procedures. For many of the indices considered, such as CAL, PPD, mMGI, PMGI and R, no intragroup differences were found: this could indicate that the control of periodontal health status is more difficult during pregnancy and a lower efficacy of the products tested could be expected, as the efficacy of paraprobiotics has been shown in previous research [37]. The absence of differences between the groups in all the periodontal indices measured could suggest that the use of the paraprobiotic toothpaste alone is sufficient to control periodontal health status and that the mousse does not provide any additional benefit. Nevertheless, a significant reduction in BOP was observed in the test group, and PCR scores were significantly reduced throughout the study period, and a high stability of recessions was observed. The present report has some limitations: A single population was analyzed, which could have excluded population-related factors. Additionally, in the present report, power calculation was set at 80% as in previous studies [61,62,63]. In the future, it could be considered to conduct further studies with greater power (example: 90–95%), thus influencing the final sample size. In addition, further reports are needed to encompass and consider further confounding factors. Another study design involving a placebo could have been drawn, but equal product without the paraprobiotic was not available and ethically questionable. Furthermore, the home treatment could have been biased by the patients themselves.

Future research should include further randomized clinical trials, possibly with longer follow-up. Assessment and improvement of pregnant women’s oral health knowledge should be evaluated [19,64]. Comparison with other commercially available paraprobiotics or other types of products could be investigated [65], as well as interaction with other preventive treatments such as ozonated gel [38], ozonated water [64] and laser therapy [66,67] or other non-surgical [68] or remineralizing [69] adjuvants. In addition, it would be interesting to test the use of specific cleaning devices such as toothbrushes [70] or interdental picks [71] to evaluate their mutual effects on periodontal parameters in pregnant women. Finally, further studies should be conducted on the use of these paraprobiotics on other categories of periodontal patients.

## 5. Conclusions

The combination of the paraprobiotic-based toothpaste and the paraprobiotic-based mousse significantly reduced BoP and plaque control over time in pregnant women, although there were no significant differences with the use of the paraprobiotic-based toothpaste alone. In addition, there was a trend towards the better stabilization of recessions after treatment with the combination of the two products.

## Figures and Tables

**Table 1:** Pregnancy period of participant at the enrollment (number and percentage on the total sample of 30 patients in brackets).

Pregnancy Period at the Enrollment (Months)	4	5	6	7	8	9
Control	1 (3.33)	2 (6.67)	2 (6.67)	4 (13.33)	3 (10.00)	3 (10.00)
Test	4 (13.33)	3 (10.00)	2 (6.67)	1 (3.33)	3 (10.00)	2 (6.67)
Total	5 (16.67)	5 (16.67)	4 (13.33)	5 (16.67)	6 (20.00)	5 (16.67)

**Table 2:** Previous pregnancies and caesarean delivery (number and percentage on the total sample of 30 patients in brackets).

Previous Pregnancies (Number)	None (0)	One (1)
Control	8 (26.67)	7 (23.33)
Test	9 (30.00)	6 (20.00)
Total	17 (56.67)	13 (43.33)
Caesarean delivery (yes/no)	Yes	No
Control	4 (13.33)	3 (10.00)
Test	2 (6.67)	4 (13.33)
Total	6 (20.00)	7 (23.33)

**Table 3:** Compositions of the tested products.

Product	Manufacturer	Composition
Biorepair^®^ Peribioma^®^ Pro	Coswell S.p.A., Funo di Argelato, BO, Italy	Bifidobacterium*, Lactobacillus*, Zinc Hydroxyapatite*, Sodium Benzoate, Glycerin, Sodium Myristoyl Sarcosinate, Pistacia Lentiscus (Mastic) Gum Oil, Aroma, Sodium Hyaluronate, Hydrated Silica, Sodium Saccharin, Cocamidopropyl Betaine, Maltodextrin, Hamamelis Virginiana Leaf Extract, Helianthus Annuus Seed Oil, Sorbitol, Spirulina Platensis Extract, Benzyl Alcohol, CI 73360, Tocopheryl Acetate, Phenoxyethanol, Calendula Officinalis Flower Extract, Silica, Limonene, Eucaliptus Globulus Leaf Oil, Retynil Palmitate, CI 77891, Ascorbic Acid, Cellulose Gum, Potassium Sorbate. *: microRepair^®^
Mousse Mouthwash Biorepair^®^ Peribioma^®^	Coswell S.p.A., Funo di Argelato, BO, Italy	Bifidobacterium*, Lactobacillus*, Zinc Hydroxyapatite*, Sodium Hyaluronate, Glycerin, Hamamelis Virginiana Leaf Extract, Eucalyptus Globulus Leaf Oil, Ascorbic Acid, Aqua, Sodium Saccharin, Calendula Officinalis Flower Extract, Pistacia Lentiscus (Mastic) Gum Oil, Tocopheryl Acetate, Maltodextrin, Phenoxyethanol, Retinyl Palmitate, Spirulina Platensis Extract, Helianthus Annuus Seed Oil, Sodium Benzoate, CI 16255, Sorbitol, Cocamidopropyl Betaine, Xylitol, Sodium Benzoate, Potassium Sorbate, PEG-40 Hydrogenated Castor Oil, Aroma, CI 16255, Limonene, BHT. *: microRepair^®^

**Table 4:** Descriptive and inferential statistics of CAL. * Means with the same letter are not significantly different (*p* > 0.05).

Group	Time	Mean	St Dev	Min	Median	Max	Significance *
Control	T0	2.63	0.44	2.02	2.57	3.60	A
	T1	2.50	0.49	2.00	2.40	3.31	A
	T2	2.62	0.46	2.00	2.66	3.26	A
	T3	2.44	0.40	2.00	2.50	3.04	A
Test	T0	2.73	0.29	2.17	2.78	3.18	A
	T1	2.90	0.26	2.45	2.84	3.29	A
	T2	2.80	0.36	2.14	2.82	3.28	A
	T3	2.66	0.43	2.05	2.60	3.36	A

**Table 5:** Descriptive statistics of PPD and inferential statistics. * Means with the same letter are not significantly different (*p* > 0.05).

Group	Time	Mean	St Dev	Min	Median	Max	Significance *
Control	T0	2.62	0.45	1.98	2.57	3.60	A
	T1	2.49	0.50	2.00	2.34	3.31	A
	T2	2.61	0.46	2.00	2.61	3.26	A
	T3	2.43	0.40	2.00	2.50	3.04	A
Test	T0	2.71	0.28	2.17	2.74	3.18	A
	T1	2.88	0.26	2.45	2.84	3.29	A
	T2	2.78	0.36	2.14	2.82	3.28	A
	T3	2.65	0.45	2.00	2.60	3.36	A

**Table 6:** Descriptive and inferential statistics of PCR. * Means with the same letter are not significantly different (*p* > 0.05).

Group	Time	Mean	St Dev	Min	Median	Max	Significance *
Control	T0	71.60	24.86	20.00	70.00	100.00	A,B
	T1	36.60	23.35	4,.00	34.00	76.00	C
	T2	28.21	23.24	0.00	20.00	75.00	C
	T3	32.67	17.43	4.00	34.50	60.00	C
Test	T0	76.07	28.99	26.00	100.00	100.00	A
	T1	53.20	23.38	10.00	50.00	100.00	A,B,C
	T2	48.67	20.99	16.00	50.00	100.00	B,C
	T3	40.54	18.89	16.00	45.00	75.00	C

**Table 7:** Descriptive statistics of the BOP and inferential statistics. * Means with the same letter are not significantly different (*p* > 0.05).

Group	Time	Mean	St Dev	Min	Median	Max	Significance *
Control	T0	47.80	37.87	1.00	45.00	100.00	A,B,C
	T1	35.33	33.55	0.00	28.00	100.00	A,C
	T2	27.50	17.92	0.00	28.00	60.00	A,C
	T3	21.92	18.61	0.00	16.00	56.00	A,C
Test	T0	68.80	37.64	5.00	100.00	100.00	B
	T1	44.13	30.05	0.00	50.00	100.00	B,C
	T2	31.33	19.10	5.00	30.00	67.00	C
	T3	31.08	29.42	5.00	20.00	100.00	C

**Table 8:** Descriptive and inferential statistics of mMGI. * Means with the same letter are not significantly different (*p* > 0.05).

Group	Time	Mean	St Dev	Min	Median	Max	Significance *
Control	T0	1.79	0.55	0.83	2.00	3.00	A
	T1	1.45	0.72	0.00	1.57	3.00	A
	T2	1.44	0.83	0.00	1.50	2.50	A
	T3	1.47	0.81	0.00	1.84	2.50	A
Test	T0	1.76	0.51	1.00	2.00	2.56	A
	T1	1.81	0.54	1.00	2.00	3.00	A
	T2	1.68	0.52	1.00	1.90	2.83	A
	T3	1.64	0.48	1.00	1.52	2.47	A

**Table 9:** Descriptive statistics of PMGI and inferential statistics. * Means with the same letter are not significantly different (*p* > 0.05).

Group	Time	Mean	St Dev	Min	Median	Max	Significance *
Control	T0	1.67	0.72	0.00	2.00	3.00	A
	T1	1.53	0.85	0.00	2.00	3.00	A
	T2	1.43	0.83	0.00	1.50	3.00	A
	T3	1.42	0.90	0.00	1.50	3.00	A
Test	T0	1.67	0.44	1.00	2.00	2.00	A
	T1	1.60	0.42	1.00	1.52	2.00	A
	T2	1.58	0.44	1.00	1.52	2.00	A
	T3	1.64	0.43	1.00	2.00	2.00	A

**Table 10:** Descriptive statistics from R and inferential statistics. * Means with the same letter are not significantly different (*p* > 0.05).

Group	Time	Mean	St Dev	Min	Median	Max	Significance *
Control	T0	0.57	0.90	0.00	0.00	2.50	A
	T1	0.64	0.89	0.00	0.00	2.50	A
	T2	0.69	0.91	0.00	0.00	2.50	A
	T3	0.80	0.94	0.00	0.50	2.50	A
Test	T0	0.58	1.08	0.00	0.00	3.33	A
	T1	0.53	0.96	0.00	0.00	2.60	A
	T2	0.52	0.93	0.00	0.00	2.40	A
	T3	0.53	1.01	0.00	0.00	2.60	A

## Data Availability

Data are available upon reasonable request to the corresponding authors.
